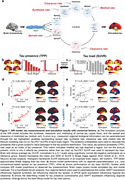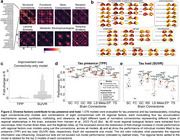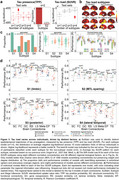# Brain connectivity drives tau presence while regional vulnerability drives tau load in Alzheimer's disease

**DOI:** 10.1002/alz70856_100814

**Published:** 2025-12-25

**Authors:** Yu Xiao, Nicola Spotorno, Lijun An, Vincent Bazinet, Justine Hansen, Olof Strandberg, Golia Shafiei, Harry H Behjat, Erik Stomrud, Ruben Smith, Sebastian Palmqvist, Rik Ossenkoppele, Niklas Mattsson‐Carlgren, Alain Dagher, Bratislav Misic, Oskar Hansson, Jacob W. Vogel

**Affiliations:** ^1^ Department of Clinical Sciences Malmö, SciLifeLab, Lund Univerisity, Lund, Sweden; ^2^ Clinical Memory Research Unit, Department of Clinical Sciences Malmö, Faculty of Medicine, Lund University, Lund, Sweden; ^3^ McConnell Brain Imaging Centre, Montréal Neurological Institute, McGill University, Montréal, QC, Canada; ^4^ Department of Psychiatry, Perelman School of Medicine, University of Pennsylvania, Philadelphia, PA, USA; ^5^ Clinical Memory Research Unit, Department of Clinical Sciences Malmö, Faculty of Medicine, Lund University, Sweden, Lund, Sweden; ^6^ Department of Neurology and Neurosurgery, McGill University, Montréal, QC, Canada; ^7^ Department of Clinical Sciences Malmö, SciLifeLab, Lund University, Lund, Sweden

## Abstract

**Background:**

Alzheimer's disease (AD) tau pathology is believed to propagate cell‐to‐cell through synaptic connections, but local properties – both intrinsic and dynamic – likely influence cellular vulnerability to tau accumulation. This study uses computational models to disentangle the roles of brain connectivity and regional vulnerability in determining where (presence) and how much (load) tau accumulates across brain regions.

**Method:**

We analyzed [18F]RO948‐PET data across 66 Desikan‐Killiany regions from 646 Aβ‐positive participants (219 unimpaired, 212 mild cognitive impairment, 215 AD dementia) from the Swedish BioFINDER‐2 study. tau‐PET standardized uptake value ratios (SUVR) represented tau load. Tau presence was calculated as tau‐PET‐positive probabilities from Gaussian mixture models (Figure 1b,1c). The Susceptible‐Infected‐Removed (SIR) model simulated regional tau synthesis, misfold, clearance, and spread over a connectome, with a priori regional information influencing model parameters. Exploratory models used eight connectome types and 49 spatial maps of brain properties (Figure 1a,2a,2b), and were fit over whole‐population and subtype‐specific tau patterns. Pearson correlation between simulated and observed tau patterns assessed model performance.

**Result:**

Tau presence aligned better with Braak staging compared to tau load. SC‐based models better explained tau presence than tau load (Figure 1d,1e), but including regional AD factors improved simulations more for tau load (Figure 1f,1g,2c). Exploratory analyses validated known mechanisms, including SC‐guided spreading influencing by MAPT (misfolding/synthesis) and Aβ (spreading), but highlighted under‐explored mechanisms like CSF clearance, developmental morphometry and various receptor distributions (Figure 2d,2e). Modeling tau propagation through receptor similarity networks yielded the best results overall. Subtype analyses reproduced known patterns for tau load patterns but no subtypes of tau presence emerged (Figure 3a,3b), suggesting consistent propagation pathways across individuals but variability in accumulation. Analyzing tau load subtypes, the MTL‐sparing subtype originated in the precuneus and propagated through functional connections, unlike other subtypes originating in the entorhinal cortex and spreading anatomically (Figure 3c,3d).

**Conclusion:**

Tau presence follows a Braak‐like pattern driven by connectivity, while tau load varies across people based on regional vulnerability. These findings validate known mechanisms but highlight under‐explored contributions of receptor distributions to tau accumulation.